# Quantitative Evaluation of Visual Aesthetics of Human-Machine Interaction Interface Layout

**DOI:** 10.1155/2020/9815937

**Published:** 2020-03-10

**Authors:** Li Deng, Guohua Wang

**Affiliations:** ^1^Key Laboratory of Oil and Gas Equipment, Ministry of Education, Southwest Petroleum University, Chengdu 610500, China; ^2^School of Mechatronic Engineering, Southwest Petroleum University, Chengdu 610500, China; ^3^State Key Laboratory of Oil and Gas Reservoir Geology and Exploitation, Southwest Petroleum University, Chengdu 610500, China

## Abstract

The current research on human-machine interaction interface layout focused on ergonomic analysis, while the research on aesthetics and aesthetic degree calculation of interface layout was insufficient. In order to objectively evaluate the aesthetic degree of interface layout, this paper put forward an aesthetic degree evaluation method of interface design based on Kansei engineering. Firstly, the perceptual image structure of interface aesthetic degree was analyzed from the perspective of aesthetic cognition. Six aesthetic image factors affecting interface aesthetic degree, including proportion, conciseness, order, rhythm, density, and equilibrium, were extracted by factor analysis method, and the variance contribution rate of each factor was taken as the weight. Secondly, according to the six aesthetic degree indexes, the calculation system of interface aesthetic degree was constructed, and the aesthetic degree value of aesthetic image factor was calculated by the corresponding aesthetic degree evaluation mathematical formula. Then, Technique for Order Preference by Similarity to Ideal Solution (TOPSIS) method was used to analyze the order of aesthetic degree superiority of design schemes, and the comprehensive aesthetic degree evaluation was carried out. Finally, the aesthetic degree evaluation of human-machine interaction interface layout of the driller's console of an AC variable frequency drilling rig was taken as an example to verify that this method was helpful for designers to optimize the design scheme. The experimental results showed that the proposed method was feasible and effective compared with the method of paired comparison commonly used in psychophysics.

## 1. Introduction

Human-machine interaction interface is a medium for people and machines to transfer information to each other. Interface layout is to arrange interface elements reasonably according to certain objective constraints, so as to ensure smooth communication between humans and machines. Interface layout, as a part of product form design, not only plays an important role in improving the ergonomic performance of products, but also has an impact on users' visual sense [1].

Traditional interface layout evaluation mainly relied on anthropometric data and three-dimensional human digital model to verify the feasibility of reachability and visibility of human-machine interaction interface. Human physiological data were collected by ergonomic hardware and device such as eye-tracking instrument [2] and analyzing the behavioral data such as learning time, error rate, and task completion time [3, 4]. For data analysis, it focused on quantitative analysis and processing of experimental data of human-machine operation. The application of aesthetics in interface design was still in its infancy, and there was no clear aesthetic standard to guide interface design.

As early as 1933, mathematician Birkhoff put forward a mathematical model of macro aesthetic feeling, expressing the “aesthetic measure” as the ratio of “order” to “complexity”, i.e., *M* = *O*/*C* [5]. The mathematical model proposed by Birkhoff is a tentative study on the fuzzy, subjective, and uncertain aesthetic feeling and thinks that aesthetic feeling can be calculated. With the improvement of users' perceptual needs, aesthetic design and aesthetic degree evaluation had become hot issues in design research [6–8]. Visual aesthetics had been shown to critically affect a variety of constructs such as perceived usability, satisfaction, and pleasure [9]. For example, Staudek [10] extended Birkhoff's aesthetic measure to formal aesthetic evaluation of regular geometrical objects, namely, Chinese vases. Ngo and Byrne [11] concerned making computers easier to learn and use by improving interface aesthetics through the use of aesthetic measures for evaluating screen layouts. Numerous researchers have explored the relationship between the golden ratio and how it relates to the human perception of aesthetics. Koh [12] asserted that golden proportion and practical geometric knowledge could be used as an extremely effective means of codifying the creative process, inspiring, and influencing creative design decisions. Wang et al. [13] put forward a feasible scheme to evaluate the aesthetic value of images according to human vision and aesthetic habits. Based on the form aesthetic principle, Chen [14] conducted aesthetic evaluation on the color, form, and decoration of furniture.

For the human-machine interaction interface design, scholars began to pay attention to the users' emotional experience and image feelings of visual aesthetics brought by interface layout [15]. The first was to study the factors that affect the aesthetic feeling of interface, and the second was to study the relationship between the layout characteristics and interface aesthetic degree. In terms of extraction and quantification of interface aesthetic factors, combining layout design features with users' visual aesthetic perception, Ngo et al. [16] put forward thirteen layout features and calculated the overall interface aesthetic degree. However, the number of features was large, the calculation was complicated, and the relationship between features was simply weighted linearly. Zhou et al. [17] extracted twelve aesthetic evaluation indexes of interface layout, studied the order of aesthetic degree superiority of design schemes by using grey correlation analysis method, and conducted comprehensive evaluation of aesthetic degree. However, the weight of three aesthetic indexes within a same factor set was the same in this study, which could not explain the influence of each evaluation index on aesthetic feeling. It did not have a strong guidance in interface improvement. In terms of constructing the mapping relationship model between interface design elements and perceptual images, Zhao et al. [18] constructed a nonlinear mapping and mathematical prediction model between interface design elements and perceptual images based on backpropagation (BP) neural network. Yun et al. [19] took software interface layout and color features as research objects and established a relationship model between features and software aesthetics by genetic algorithm and radial basis function (RBF) network. However, these studies were built on the basis of few samples of specific typical cases, and it remained to be seen whether their prediction accuracy and research conclusions could be extended to other interface designs.

It could be seen that, with the development of Kansei engineering, aesthetic principles such as aesthetic measurement, the golden ratio, and form aesthetic principle had been successively applied to aesthetic design and evaluation of product form [20, 21], product color [22], plane graphic [23], web page [24], and so on. There were a lot of theoretical foundations for aesthetic research, but the scholars' studies on interface layout aesthetics were scattered, and the links between various studies were not close enough [25]. Therefore, on the basis of previous studies, this paper attempted to use Kansei engineering's quantitative analysis method of perceptual experience [26, 27], extract aesthetic image factors of human-machine interaction interface from the perspective of aesthetic cognition, construct the perceptual image structure of aesthetic degree, and establish the corresponding mathematical formula of aesthetic evaluation to calculate the aesthetic value of aesthetic image factors. At last, the Technique for Order Preference by Similarity to Ideal Solution (TOPSIS) method would be used to rank the aesthetic degree of schemes and assist the designer in the layout analysis and design of human-machine interaction interface.

## 2. Methodological Framework of This Paper

**Figure 1:**
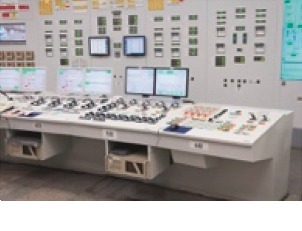
The methodological framework of this paper.

The specific research framework is shown in Figure 1.

**Step 1 .**

Using the method of Kansei engineering to study the users' perceptual image, the factors that affect the aesthetic degree of human-machine interaction interface are described, and the aesthetic image factors are extracted by factor analysis method to construct the perceptual image structure of interface aesthetic degree.

**Step 2 .**

The characteristics of layout interface and layout object are analyzed, the influence of layout objects' location distribution on visual aesthetics is studied, and the aesthetic degree calculation formula of aesthetic image factors is constructed.

**Step 3 .**

The TOPSIS method is used to analyze the aesthetic value of layout design scheme, and the comprehensive evaluation value is calculated to rank interface aesthetic degree.

**Step 4 .**

An example is given to illustrate the implementation steps of the evaluation method of interface aesthetic degree, and the effectiveness of the proposed method is verified.

## 3. Image Extraction of Visual Aesthetic Elements

In the process of traditional human-machine interaction interface layout design, designers often design under the constraints of layout interface space and functions of the objects to be laid out according to their own experience. Due to the lack of communication with end users, such design results may meet the geometric constraints and functional requirements, but may be subjective in the aesthetic experience of designers and may deviate from the users' perceptual needs and affect the user experience [17]. Therefore, at the beginning of interface layout design, it is necessary to deeply study the users' perception mechanism and analyze the perceptual image structure of interface aesthetic degree, so as to improve the explanatory effectiveness of the evaluation method of interface aesthetic degree, which is also the theoretical basis of the aesthetic degree calculation system.

The pictures of human-machine interaction interface of various products were collected extensively, and these pictures were preliminarily classified. The pictures of similar types were removed. Finally, ten representative samples of human-machine interaction interface were selected [28, 29]. As shown in Table 1, the sample includes product interfaces of different specifications and types such as military, civil, mechanical equipment, daily necessities, and plane layout. Image vocabularies were used to express the aesthetic preference of human-machine interaction interface layout, and a large number of image vocabularies suitable for the aesthetic image expression of human-machine interaction interface were collected through network, literature, interviews, and other ways [30]. The repeated meaning vocabularies were deleted, and the similar meaning vocabularies were merged. Thirty-three vocabularies reflecting the aesthetic image characteristics of the interface were screened out. The aesthetic image of interface layout reflects users' perceptual perception and needs, while perceptual needs have the characteristics of fuzziness, dynamism, complexity, and inducibility. In order to avoid the cognitive differences caused by personal understanding differences, some abstract perceptual descriptive vocabularies were avoided. Five industrial design teachers and fifteen industrial design students conducted image analysis on the samples. By using Likert scale, five-point scale was used to distinguish the subjects' recognition of these image vocabularies to describe the interface aesthetic degree. The subjects' score constituted the state degree set *V*, *V* = {Very not 1 Comparison not 2 General 3 Comparison 4 Very 5}. The higher the score, the closer the feeling described by the image vocabularies; for example, for “proportion,” a very bad proportion was 1 point, a little bad was 2 points, a neutral attitude was 3 points, a little good was 4 points, and a very good was 5 points.

If there were too many image vocabularies, it would not be conducive to study and interpret user images, and increasing the cognitive burden of interface aesthetic evaluation. Therefore, factor analysis was used to extract a few integrated image vocabularies to reduce the cognitive dimension. Factor analysis is a multivariate statistical analysis method that studies how to condense many original variables into a few factors with the least information loss and how to make factors have a certain explanatory naming. In factor analysis, it is necessary to first study the analysis conditions of factors, that is, whether the original variables are correlated or not. Therefore, firstly, Kaiser–Meyer–Olkin (KMO) test was used to measure whether there was correlation between variables for factor analysis. The closer the KMO value was to 1, the stronger the correlation between variables was, and the original variables were more suitable for factor analysis. Generally, according to Kaiser's KMO metric, this analysis method could be adopted when the KMO measure was greater than 0.7. Then, the Bartlett test of sphericity was used to test the hypothesis that the correlation coefficient matrix was a unit matrix. If the original hypothesis could not be rejected, it could be considered that there was no significant difference between the correlation coefficient matrix and the unit matrix; then, the original variables were not suitable for factor analysis.

On the basis of questionnaire survey, the scoring data obtained from the scale were imported into SPSS Statistics software for factor analysis. The key to factor analysis was to solve the factor loading matrix based on the sample data, which was solved by the most widely used principal component analysis method. As shown in Table 2, six visual aesthetic image factors are extracted, and the cumulative variance contribution rate reaches 86.980%, which meet the principle of “generally select the number of eigenvalues when the cumulative variance contribution rate is greater than 0.85 as the number of factors.” In this way, thirty-three variables were reduced to six factors, and most information of the original variables could be reflected.

As shown in Table 3, six image factors are extracted from thirty-three image vocabularies, and the perceptual image structure of interface aesthetic degree is established. The following is the explanation and analysis of factors.

*Image factor 1: Proportion*. Golden section, uniformity, and other four image vocabularies have a high load on the first factor, and the first factor mainly explains these four variables, which can be explained as proportion. Proportion can be understood as that the relative measurement relationship between the length and width of a layout object. Proportion is rational, and if the proportion of layout object is close to the common aesthetic proportion, such interface design can meet the aesthetic needs of most people.

*Image factor 2: Conciseness*. Simple, concise, and other seven image vocabularies have a high load on the second factor, and the second factor mainly explains these seven variables, which can be explained as conciseness. The layout objects in a same system should be standardized and aligned as much as possible. Without affecting the effective transmission of information, the complexity and variability of the form of layout objects should be reduced. The interface layout design should be concise and clear, so as to avoid too much burden on cognition and memory to users.

*Image factor 3: Order*. Sequence, ease, and other five image vocabularies have a high load on the third factor, and the third factor mainly explains these five variables, which can be explained as order. According to the laws of human visual perception, the differentiated layout of interface elements makes the interface layout design follow certain laws of line of sight induction, which effectively improves the users' accuracy of visual recognition and operation performance.

*Image factor 4: Rhythm*. Harmony, cycle, and other seven image vocabularies have a high load on the fourth factor, and the fourth factor mainly explains these seven variables, which can be explained as rhythm. Cadence and rhythm are inseparable unity; they are the common language of aesthetic feeling. The beauty of cadence and rhythm is a form of repetition, continuity, and change, which can be realized through the changes of size, position, and density of the layout objects in layout design.

*Image factor 5: Density*. Concentration, dense, and other five image vocabularies have a high load on the fifth factor, and the fifth factor mainly explains these five variables, which can be explained as density. Pursuing the reasonable application of layout interface area, the sense of looseness, and oppression, respectively, brought by sparse arrangement and excessive compactness should be avoided. The ideal layout is relaxed and balanced and making reasonable use of space.

*Image factor 6:* Equilibrium. Balance, symmetry, and other five image vocabularies have a high load on the sixth factor, and the sixth factor mainly explains these five variables, which can be explained as equilibrium. The whole visual information of interface should be balance and stability. Pursuing the rationality of the size and distribution of interface elements and the visual fatigue and information loss caused by the imbalance of information layout should be avoided.

## 4. Aesthetic Degree Calculation of Visual Aesthetic Image Factor

**Figure 2:**
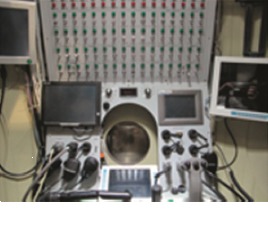
The abstract representation of interface layout.

In order to quantify the visual aesthetics of interface layout accurately, as shown in Figure 2, the layout interface is abstracted as rectangle, and the layout objects are abstracted as rectangle or circle. The layout interface was divided into four areas: top left, top right, bottom left, and bottom right. Combining with the visual aesthetic image structure of interface shown in Table 3, six formulas for calculating the aesthetic degree of image factors were constructed, respectively. The aesthetic degree calculation of order, rhythm, and equilibrium involved the area and location of layout objects in these four areas. Detailed calculation is as follows.

### 4.1. Proportion *M*_1_

Proportion refers to the coordinated relationship between the layout object itself and the layout interface of human-machine interaction interface. Proportion, as one of the form aesthetic principles, is an aesthetic measurement relationship created by people in long-term life practice. It is a theory to express the beauty of modern life and modern science and technology by ratio. The commonly used aesthetic proportions are 1 : 1, 1 : 1.414, 1 : 1.618, 1 : 1.732, and 1 : 2. Therefore, the proportion was defined as calculating the similarity of the ratio value between the aesthetic proportions and the proportional relation of layout object and layout interface:

(1) $$\begin{array}{c} M_{1}=\frac{\left|P_{\text{object}}\right|+\left|P_{\text{interface}}\right|}{2}, \end{array}$$

where

(2) $$\begin{array}{c} P_{\text{object}}=\frac{1}{n}\sum_{i}^{n}\left(1-\frac{\min\left(\left|P_{j}-P_{\text{object},i}\right|\right)}{0.5}\right), \end{array}$$

(3) $$\begin{array}{c} P_{\text{interface}}=1-\frac{\min\left(\left|P_{j}-P_{\text{interface}}\right|\right)}{0.5}, \end{array}$$

(4) $$\begin{array}{c} P_{\text{object},i}=\left\{\begin{array}{cc} \frac{h_{i}}{b_{i}}, & b_{i}\geq h_{i},\\ \frac{b_{i}}{h_{i}}, & h_{i}\geq b_{i}, \end{array}\right. \end{array}$$

(5) $$\begin{array}{c} P_{\text{interface}}=\left\{\begin{array}{cc} \frac{h_{\text{interface}}}{b_{\text{interface}}}, & b_{\text{interface}}\geq h_{\text{interface}},\\ \frac{b_{\text{interface}}}{h_{\text{interface}}}, & h_{\text{interface}}\geq b_{\text{interface}}, \end{array}\right. \end{array}$$

(6) $$\begin{array}{c} P_{j}=\left\{\frac{1}{1},\;\frac{1}{1.414},\frac{1}{1.618},\frac{1}{1.732},\frac{1}{2}\right\}, \end{array}$$

where *P*_object_ represents the proportional relation of layout object; *P*_interface_ represents the proportional relation of layout interface; *h*_*i*_  and *b*_*i*_ represent the height and width of layout object, respectively. *h*_interface_ and *b*_interface_ represent the height and width of layout interface, respectively. *P*_*j*_ represents the commonly used proportion.

### 4.2. Conciseness *M*_2_

Conciseness refers to the fact that the layout objects on the human-machine interaction interface have fewer forms and styles, the layout elements with the same size appear repeatedly, and the central position of the layout objects is on a horizontal or vertical line. Such arrangement can easily achieve a unified and concise visual effect. Therefore, conciseness was defined as calculating the consistency, alignment, and combination degree of the size of layout objects, using as few presentation elements as possible to convey the corresponding information, and reducing the difficulty for users to understand the formal meaning of interface design:

(7) $$\begin{array}{c} M_{2}=\frac{\left(3/\left(n_{h}+n_{v}+n\right)\right)+\left(1-\left(n_{\text{type}}/n\right)\right)}{2}, \end{array}$$

where *n*_*h*_ and *n*_*v*_ represent the number of alignment points of layout objects in the horizontal and vertical directions, respectively; *n*_type_ represents the number of types of layout objects; *n* represents the total number of layout objects.

### 4.3. Order *M*_3_

The human eye has the characteristics of visual movement from left to right and from top to bottom. The line of sight often moves from the layout object with large area to the layout object with small area. The interface layout that conforms to this visual patrol rule can effectively guide the users' observation sequence. Therefore, the sense of order was defined as expressing the information measurement described above by the following mathematical formula:

(8) $$\begin{array}{c} M_{3}=1-\frac{\sum_{j=\text{TL},\text{TR},\text{BL},\text{BR}}\left|S_{j}-T_{j}\right|}{8}\;,\;\left\{S_{\text{TL}},S_{\text{TR}},S_{\text{BL}},S_{\text{BR}}\right\}=\left\{4,3,2,1\right\}, \end{array}$$

(9) $$\begin{array}{c} T_{j}=\left\{\begin{array}{cc} 4, & \text{if }w_{j}=\max\text{ in }w,\\ 3, & \text{if }w_{j}=2\text{nd in }w,\\ 2,\; & \text{if }w_{j}=3\text{rd in }w,\\ 1, & \text{if }w_{j}=\min\text{ in }w, \end{array}\right.\;j=\text{TL,TR,BL,BR,} \end{array}$$

(10) $$\begin{array}{c} w_{j}=S_{j}\sum_{i}^{n_{j}}a_{ij},\;j=\text{TL},\text{TR},\text{BL},\text{BR,} \end{array}$$

where TL, TR, BL, and BR represent the top left, top right, bottom left, and bottom right areas of human-machine interaction interface, respectively; *a*_*ij*_ represents the area of layout object *i* in the layout interface area *j*; *S*_*j*_ represents the weight of the layout interface area *j*.

### 4.4. Rhythm *M*_4_

Rhythm is a regular and periodically changing form of movement, which is formed by regular repetition in design. In the human-machine interaction interface layout design, the size, number, arrangement, and form of the layout objects can arouse users' physiological feelings, psychological emotional activities, and aesthetic feeling of rhythm. Therefore, rhythm was defined as calculating the difference of position and area of layout objects in these four areas: top left, top right, bottom left, and bottom right areas of layout interface:

(11) $$\begin{array}{c} M_{4}=1-\frac{\left|R_{x}\right|+\left|R_{y}\right|+\left|R_{\text{area}}\right|}{3}, \end{array}$$

(12) $$\begin{array}{c} R_{x}=\frac{\left(\left|X_{\text{TL}}^{\prime }-X_{\text{TR}}^{\prime }\right|+\left|X_{\text{TL}}^{\prime }-X_{\text{BL}}^{\prime }\right|+\left|X_{\text{TL}}^{\prime }-X_{\text{BR}}^{\prime }\right|+\left|X_{\text{TR}}^{\prime }-X_{\text{TL}}^{\prime }\right|+\left|X_{\text{TR}}^{\prime }-X_{\text{BL}}^{\prime }\right|+\left|X_{\text{TR}}^{\prime }-X_{\text{BR}}^{\prime }\right|\right)}{6}, \end{array}$$

(13) $$\begin{array}{c} R_{y}=\frac{\left(\left|Y_{\text{TL}}^{\prime }-Y_{\text{TR}}^{\prime }\right|+\left|Y_{\text{TL}}^{\prime }-Y_{\text{BL}}^{\prime }\right|+\left|Y_{\text{TL}}^{\prime }-Y_{\text{BR}}^{\prime }\right|+\left|Y_{\text{TR}}^{\prime }-Y_{\text{TL}}^{\prime }\right|+\left|Y_{\text{TR}}^{\prime }-Y_{\text{BL}}^{\prime }\right|+\left|Y_{\text{TR}}^{\prime }-Y_{\text{BR}}^{\prime }\right|\right)}{6}, \end{array}$$

(14) $$\begin{array}{c} R_{\text{area}}=\frac{\left(\left|A_{\text{TL}}^{\prime }-A_{\text{TR}}^{\prime }\right|+\left|A_{\text{TL}}^{\prime }-A_{\text{BL}}^{\prime }\right|+\left|A_{\text{TL}}^{\prime }-A_{\text{BR}}^{\prime }\right|+\left|A_{\text{TR}}^{\prime }-A_{\text{TL}}^{\prime }\right|+\left|A_{\text{TR}}^{\prime }-A_{\text{BL}}^{\prime }\right|+\left|A_{\text{TR}}^{\prime }-A_{\text{BR}}^{\prime }\right|\right)}{6}, \end{array}$$

(15) $$\begin{array}{c} X_{j}=\sum_{i}^{n_{j}}\left|x_{ij}-x_{c}\right|, \end{array}$$

(16) $$\begin{array}{c} Y_{j}=\sum_{i}^{n_{j}}\left|y_{ij}-y_{c}\right|, \end{array}$$

(17) $$\begin{array}{c} A_{j}=\sum_{i}^{n_{j}}a_{ij}, \end{array}$$

(18) $$\begin{array}{c} j=\text{TL},\text{TR},\text{BL},\text{BR,} \end{array}$$

(19) $$\begin{array}{c} E_{i}^{\prime }=\frac{E_{i}-\min_{1\leq j\leq n}E_{j}}{\max_{1\leq j\leq n}E_{j}-\min_{1\leq j\leq n}E_{j}},\;\;\;E=X,\;Y,\;A, \end{array}$$

where *X*_*j*_′, *Y*_*j*_′, and *A*_*j*_′ represent the dimensionless values after normalization of *X*_*j*_, *Y*_*j*_, and *A*_*j*_, respectively; TL, TR, BL, and BR represent the top left, top right, bottom left, and bottom right areas of human-machine interaction interface, respectively; (*x*_*ij*_,  *y*_*ij*_) and (*x*_*c*_,  *y*_*c*_) represent the coordinates of center position of layout object and layout interface, respectively; *a*_*ij*_ represents the area of layout object *i* in the layout interface area *j*; *n*_*j*_ represents the number of layout objects in quadrant *j*.

### 4.5. Density *M*_5_

Density refers to the density degree of layout objects arranged in a layout interface. According to the existing research findings, it is quite appropriate that the interface with a density degree of about 50% is neither too tight nor too loose. Therefore, density was defined as calculating the difference between the actual density degree and the optimal density degree of interface layout:

(20) $$\begin{array}{c} M_{5}=1-2\left|0.5-\frac{\sum_{i}^{n}a_{i}}{a_{\text{interface}}}\right|, \end{array}$$

where *a*_*i*_ represents the area of layout object; *a*_interface_ represents the area of layout interface; *n* is the number of layout objects. In the formula, the optimal density level of interface layout was set as 50%.

### 4.6. Equilibrium *M*_6_

Equilibrium refers to the relative volume relationship between the top and bottom parts and the left and right parts of human-machine interaction interface. The large areas are heavy and small areas are light in visual sense. In the layout design, the sum of the volume moment on both sides of the horizontal and vertical directions should be equal, so as to achieve a rough visual balance. Therefore, equilibrium was defined as calculating the difference between the volume moment of the objects on both sides of the horizontal and vertical axes of the visual center of gravity:

(21) $$\begin{array}{c} M_{6}=1-\frac{\left(\left|\left(w_{L}-w_{R}\right)/\left(\max\left(\left|w_{L}\right|,\left|w_{R}\right|\right)\right)\right|+\left|\left(w_{T}-w_{B}\right)/\left(\max\left(\left|w_{T}\right|,\left|w_{B}\right|\right)\right)\right|\right)}{2}, \end{array}$$

(22) $$\begin{array}{c} w_{j}=\sum_{i}^{n_{j}}a_{ij}d_{ij},\;j=L,R,T,B, \end{array}$$

where *L*, *R*, *T*, and *B* represent the left, right, top, and bottom quadrants of the interface, respectively; *a*_*ij*_ represents the area of layout object *i* in quadrant *j*; *d*_*ij*_ represents the distance between the layout object *i* and the axis of visual center of gravity; *n*_*j*_ represents the number of layout objects in quadrant *j*.

## 5. Comprehensive Aesthetic Degree Evaluation Based on TOPSIS Method

In the visual aesthetics evaluation of human-machine interaction interface layout, multiple decision-makers should evaluate several schemes based on aesthetic evaluation indexes, which belonged to multiattribute decision-making problem. TOPSIS method is a mature and effective method in multiattribute decision-making. It constructs the positive and negative ideal solutions of multiple indexes and then determines the distance between the object to be evaluated and the positive and negative ideal solutions to judge the merits and demerits of the evaluation results. TOPSIS method makes full use of the original data information, and the calculation is simple and easy. The results can accurately reflect the gap between the evaluation schemes. This method is especially suitable for the aesthetic degree evaluation of associated schemes and derivative schemes.

In the aesthetic degree evaluation of human-machine interaction interface layout, *m* aesthetic degree evaluation indexes for *n* layout schemes constitute the initial matrix *A* of *n* × *m*:

(23) $$\begin{array}{c} A=\left[\begin{array}{cccc} a_{11} & a_{12} & \cdots & a_{1m}\\ a_{21} & a_{22} & \cdots & a_{2m}\\ \cdots & \cdots & \cdots & \cdots \\ a_{n1} & a_{n2} & \cdots & a_{nm} \end{array}\right]. \end{array}$$

The layout scheme set is *A* = (*A*_1_, *A*_2_, ..., *A*_n_), *n* ≥ 3, and the attribute vector (aesthetic degree evaluation index) of each scheme is *X* = {*X*_1_, *X*_2_, ..., *X*_*m*_}. The *m* attribute values of each scheme *A*_*i*_ (*i* = 1, 2, ..., *n*) in scheme set *A* constitute the vector *x* = {*x*_1_, *x*_2_, ..., *x*_*m*_}. The positive ideal solution *V*^+^ is the best scheme envisaged in scheme set *A*, and each attribute value of which is the best value among alternative schemes, while the negative ideal solution *V*^−^ is the worst scheme envisaged in scheme set *A*, and each attribute value of which is the worst value among alternative schemes. Then, the closeness degree of each layout scheme to the ideal scheme is calculated. By measuring the difference between the aesthetic degree of each layout scheme and the best aesthetic degree, the priority of the layout scheme is determined. The larger the closeness degree value is, the better the corresponding layout scheme is.

The steps of TOPSIS method are as follows:

1. The normalized decision matrix *B*_*ij*_={*b*_*ij*_} is obtained by normalizing the decision matrix through the vector normalization method:

(24) $$\begin{array}{c} b_{ij}=\frac{a_{ij}}{\sqrt{\sum_{i=1}^{n}a_{ij}^{2}}}. \end{array}$$

(2) The weighted normal matrix *C* is constructed according to the weight vector. Considering the relationship of each factor comprehensively, the weight is determined according to the initial information provided by each factor, that is, the variance contribution rate of each factor in Table 2 is taken as the weight to eliminate the influence of subjective factors:

(25) $$\begin{array}{c} C_{ij}=\left\{c_{ij}\right\}=\left\{w_{j}b_{ij}\right\}=\left[\begin{array}{cccc} w_{1}b_{11} & w_{2}b_{12} & \cdots & w_{m}b_{1m}\\ w_{1}b_{21} & w_{2}b_{22} & \cdots & w_{m}b_{2m}\\ \cdots & \cdots & \cdots & \cdots \\ w_{1}b_{n1} & w_{2}b_{n2} & \cdots & w_{m}b_{nm} \end{array}\right]. \end{array}$$

(3) Determine the positive ideal solution and negative ideal solution. The evaluation indexes of interface aesthetic degree are all benefit indexes. For example, the *j*th value of positive ideal solution *V*^+^ is *v*_*j*_^+^, and the *j*th value of negative ideal solution *V*^−^ is *v*_*j*_^−^:

(26) $$\begin{array}{c} V_{j}^{+}=\underset{1\leq i\leq n}{\max}c_{ij},\\ V_{j}^{-}=\underset{1\leq i\leq n}{\min}c_{ij}. \end{array}$$

(4) Calculate the distance from each scheme to positive and negative ideal solutions. The distance from each aesthetic degree evaluation index to positive ideal solution and negative ideal solution is set as *d*_*i*_^+^ and *d*_*i*_^−^ (*i* = 1, 2, ..., *n*), respectively:

(27) $$\begin{array}{c} d_{i}^{+}=\sqrt{\sum_{j=1}^{m}{\left(c_{ij}-v_{j}^{+}\right)}^{2},}\\ d_{i}^{-}=\sqrt{\sum_{j=1}^{m}{\left(c_{ij}-v_{j}^{-}\right)}^{2}.} \end{array}$$

(5) Calculate the comprehensive evaluation index. The closeness degree from each scheme to ideal scheme is set as *d*_*i*_, and the order of advantages and disadvantages of the schemes can be determined according to the value of *d*_*i*_:

(28) $$\begin{array}{c} d_{i}=\frac{d_{i}^{-}}{d_{i}^{-}+d_{i}^{+}}. \end{array}$$

## 6. Case

**Figure 3:**
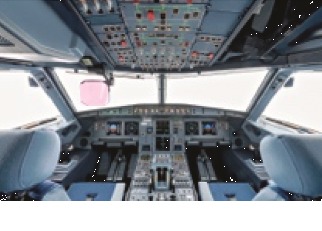
Interface layout design scheme.

This paper illustrated the quantitative evaluation process of visual aesthetics by taking the human-machine interaction interface layout aesthetic degree evaluation of the driller's console of an AC variable frequency drilling rig as an example.  Figure 3 shows four design schemes of interface layout. In order to quantify the aesthetics of interface layout, the interface to be evaluated was simplified to a plane layout problem in the preprocessing stage. The layout interface was set as rectangular, and the layout object was set as rectangular or circular. Its specific size is shown in Table 4.

The twelve layout objects were abstracted into two categories: rectangle and circle. There were four sizes of circle. The layout objects were purchased instruments, and their size and shape were fixed and could only be laid out in position. Taking into account the size of the instrument and the distance between installations, the layout should be as compact as possible. Because if the longer and larger the layout interface was, the larger the angle of eye movement and head rotation was; when the driller was observing, the more inconvenient it was to observe.

From the perspective of ergonomics, we should refer to the visual field of human eyes and the characteristics of the visual area and arrange all kinds of instruments according to their importance to facilitate observation. According to the survey results, the most frequently observed instrument was weight indicator. Secondly, the parameters instrument and the stand pipe pressure gauge were observed more frequently. Then, the three disc brake pressure instruments of left clamp pressure gauge, right clamp pressure gauge, and safety clamp pressure gauge, which were more frequently observed and more important than other small instruments. The observation of rotary torque meter and crabs torque meter was relatively less. Air pressure, rotary oil pressure gauge, and winch oil pressure gauge were small instruments with low importance. The observation times of liquid hydraulic pressure gauge were the least.

The layout interface of scheme 1, scheme 2, and scheme 3 was the same size, while the length of the layout interface of scheme 4 was longer but with the same width. The layout objects of the four schemes were in different positions. In scheme 1, the weight indicator with the most observations was arranged in front of the observer, and then other display devices were arranged to the left and right sides, respectively, based on the center line of the weight indicator. The parameters instrument was arranged on the left side of the weight indicator. The stand pipe pressure gauge was arranged on the right side of the weight indicator. The three disc brake pressure instruments were frequently observed and arranged in front of the human body for quick finding. According to people's cognitive habits, the left clamp pressure gauge and the right clamp pressure gauge were, respectively, arranged at the bottom left and the bottom right of the weight indicator, and the safety clamp pressure gauge was arranged at the right side of the right clamp pressure gauge. The observation of rotary torque meter and crabs torque meter was relatively less, and the dial belonged to medium instrument, which was arranged on the far right side. Air pressure, rotary oil pressure gauge, and winch oil pressure gauge were arranged in the right space of weight indicator. The observation frequency of the liquid hydraulic pressure gauge was not much, which was arranged between the safety clamp pressure gauge and crabs torque meter. On the basis of scheme 1, the instruments on the left and right sides of scheme 2 were exchanged with the weight indicator as the center. On the basis of scheme 2, some instruments on the upper and lower sides of scheme 3 were interchanged. In scheme 4, based on scheme 1, the positions of the six instruments on the right side were adjusted to make the arrangement sparse and orderly. Overall, the layout of scheme 1 and scheme 4 was more in line with ergonomic.

### 6.1. Aesthetic Degree Calculation

According to the aesthetic degree calculation formulas (1)–(22) in Section 4, the quantitative index values of interface aesthetics of the four schemes were calculated, respectively.  Table 5 shows the calculation results of six aesthetic degree index values of four schemes.

According to the data in Table 5, since the size and shape of twelve layout objects in each layout scheme were fixed and only the position was changed, so there was no difference in the proportion of scheme 1, scheme 2, and scheme 3. The length of layout interface in scheme 4 was the longest, and its proportion (0.78991) got worse, but the density (0.94997) of layout was the best. The number and type of layout objects in these four schemes were the same, only that the alignment degree of layout objects was different, so the conciseness of these four schemes was not significantly different. The sense of order formed by the law of visual movement from left to right and from top to bottom was the best. And it was calculated that the order of visual guidance of scheme 1 and scheme 4 was top left, bottom left, top right, and bottom right, the order of visual guidance of scheme 2 and scheme 3 was top right, top left, bottom left, and bottom right, and the order score of these four schemes was consistent. Scheme 1, scheme 2, and scheme 4 all reflected the change of rhythm and the sense of equilibrium of the interface, while scheme 3 was slightly inferior (0.36705 and 0.53646). In general, since the size of the layout interface had only two specifications, and the size of the layout objects had not changed, so the aesthetic degree index of these four schemes was relatively close and the difference was not significant. Scheme 3 had the worst sense of rhythm and equilibrium and should be eliminated first. While the other three schemes were too close to each other, TOPSIS method should be used for further comparative analysis.

### 6.2. Comprehensive Evaluation

Based on the values of aesthetic degree evaluation index of the four layout schemes shown in Table 5, the order of pros and cons of the schemes could be calculated according to the TOPSIS method introduced in Section 5. The steps were as follows:

1. Four layout schemes and six aesthetic degree evaluation indexes constituted the initial matrix *A* of 4 × 6:

(29) $$\begin{array}{c} A=\left[\begin{array}{cccccc} 0.81670 & 0.34722 & 0.75000 & 0.45897 & 0.85732 & 0.69968\\ 0.81670 & 0.34722 & 0.75000 & 0.45297 & 0.85732 & 0.69968\\ 0.81670 & 0.34167 & 0.75000 & 0.36705 & 0.85732 & 0.53646\\ 0.78991 & 0.34722 & 0.75000 & 0.49936 & 0.94997 & 0.60830 \end{array}\right]. \end{array}$$

(2) Formula (24) was used to normalize matrix *A* into matrix *B*:

(30) $$\begin{array}{c} B=\left[\begin{array}{cccccc} 0.50408 & 0.50200 & 0.50000 & 0.51317 & 0.48634 & 0.54687\\ 0.50408 & 0.50200 & 0.50000 & 0.50646 & 0.48634 & 0.54687\\ 0.50408 & 0.49396 & 0.50000 & 0.41039 & 0.48634 & 0.41930\\ 0.48755 & 0.50200 & 0.50000 & 0.55833 & 0.53890 & 0.47545 \end{array}\right]. \end{array}$$

(3) The weighted matrix *C* was calculated by the normalized matrix *B*. The variance contribution rate of factors was taken as the weight. The weight of each aesthetic degree evaluation index is shown in Table 6:

(31) $$\begin{array}{c} C=\left[\begin{array}{cccccc} 0.09024 & 0.05245 & 0.03832 & 0.09070 & 0.07716 & 0.09531\\ 0.09024 & 0.05245 & 0.03832 & 0.08952 & 0.07716 & 0.09531\\ 0.09024 & 0.05161 & 0.03832 & 0.07254 & 0.07716 & 0.07308\\ 0.08728 & 0.05245 & 0.03832 & 0.09869 & 0.08550 & 0.08286 \end{array}\right]. \end{array}$$

(4) Determine the positive ideal solution *V*_*j*_^+^ and the negative ideal solution *V*_*j*_^−^:

(32) $$\begin{array}{c} V_{j}^{+}=\left[\begin{array}{cccccc} 0.09024 & 0.05245 & 0.03832 & 0.09869 & 0.08550 & 0.09531 \end{array}\right],\\ V_{j}^{-}=\left[\begin{array}{cccccc} 0.08728 & 0.05161 & 0.03832 & 0.07254 & 0.07716 & 0.07308 \end{array}\right]. \end{array}$$

(5) Calculate the distance *d*_*i*_^+^ and *d*_*i*_^−^ from each scheme to positive ideal solution and negative ideal solution:

(33) $$\begin{array}{c} d_{1}^{+}=0.01154,\\ d_{2}^{+}=0.01239,\\ d_{3}^{+}=0.03533,\\ d_{4}^{+}=0.01279,\\ d_{1}^{-}=0.02888,\\ d_{2}^{-}=0.02814,\\ d_{3}^{-}=0.00296,\\ d_{4}^{-}=0.02915. \end{array}$$

(6) Calculate the closeness degree *d*_*i*_ of each scheme to the ideal scheme:

(34) $$\begin{array}{c} d_{1}=0.71439,\\ d_{2}=0.69428,\\ d_{3}=0.07731,\\ d_{4}=0.69496. \end{array}$$

According to the closeness degree *d*_*i*_, the order of the aesthetic degree of the layout scheme was arranged from the largest to the smallest. Therefore, the aesthetic degree ranking of these four schemes was scheme 1 > scheme 4 > scheme 2 > scheme 3.

### 6.3. Conclusion Verification

Based on the aesthetic cognition and visual perception principles, this paper combined the perceptual image structure of interface aesthetics to establish the aesthetic calculation formula of the corresponding index, reflected the fuzzy sensibility with mathematical logic, and analyzed the relationship between the human-machine interaction interface layout and the user's perception of aesthetic degree with objective numerical calculation method. In view of the fact that aesthetic cognition was a complex and subjective psychological activity, the aesthetic degree of the four layout schemes was evaluated subjectively by the method of paired comparison commonly used in psychophysics in this paper. The four schemes in Figure 3 were successively numbered as A, B, C, and D. After matching the schemes, the samples were presented by E-prime software. Each scheme should be compared with the other schemes separately, and the number of pairs was 4^∗^ (4 − 1)/2 = 6, that is, six times should be compared and chosen in one round of experiments. In order to eliminate the sequence errors, two rounds of comparison were conducted. The position of the picture presented in the second round was exchanged with the position of the picture in the first round, so as to eliminate the influence of the left and right order on psychological perception. Two rounds of experiments were conducted for a total of 12 times. The sequence of picture presentation in the experiment is shown in Table 7.

According to the scheduled order in Table 7, pairs of pictures were presented on the left and right sides of the screen. Subjects selected one of the pictures that they thought was beautiful according to their subjective intuition and repeated it until they completed twelve times of selection. Five students majoring in industrial design were invited to participate in this experiment. The original data selected by each subject in the experiment are shown in Table 8. From the statistical data in Table 9, it can be seen that scheme 1 has been selected the most times, followed by scheme 4, scheme 2, and scheme 3. This order was consistent with that obtained by TOPSIS method on the basis of aesthetic degree calculation. This showed that the proposed quantitative method of visual aesthetics could simulate the users' psychological evaluation mechanism effectively and play a guiding role in the aesthetic design of human-machine interaction interface.

## 7. Discussion

The subtle adjustment of the spatial position of interface elements could often create a very obvious sense of visual difference, thus affecting the aesthetic experience of users. Users and designers could use the image of Kansei engineering to express the aesthetic feeling of human-machine interaction interface. In this paper, the aesthetic image factors that affect the aesthetic feeling of the interface were extracted, the aesthetic calculation formula of the corresponding index was established, and the comprehensive evaluation value of the aesthetic degree of the layout scheme was obtained by TOPSIS method. The method proposed in this paper provided an auxiliary means for the evaluation of interface layout design, which had a certain practical significance. However, there are still some problems need to be further discussed and studied.

First, most of the traditional aesthetic theory was the experience summary and abstract induction of the form aesthetic principle, and these qualitative analyses could not accurately reveal the aesthetic degree of human-machine interaction interface, lacking quantitative research on the mathematical relationship. In this paper, six aesthetic image factors were extracted by factor analysis, and the aesthetic value of aesthetic image factors was calculated by corresponding aesthetic calculation formula. We believe that user perception of interface aesthetics can be objectively quantified by the theory and method of Kaisei engineering, which is consistent with the views of Ngo et al. [16] and Zhou et al. [17]. The difference is that Ngo et al. put forward thirteen aesthetic degree indexes, while Zhou et al. put forward twelve aesthetic degree indexes on the basis of Ngo, all of which are complicated in calculation. In the study of Zhou et al., the weights of the three aesthetic degree indexes in the same factor are set to be the same. In this paper, the variance contribution rate of the factor is taken as the weight, which can reflect the influence degree of each evaluation index on the overall aesthetic feeling. However, there are many methods to determine the weight, so whether other weighting methods should be used remains to be further compared and studied.

Second, Sonderegger and Sauer [15] found that the visual appearance of mobile phones had a positive effect on performance. Aesthetic factors are very important for human-machine interaction interface design. The method proposed in this paper was helpful for designers to find a human-machine interaction interface meeting the requirements of visual aesthetics. However, does the human-machine interaction interface that meets the aesthetic requirements also have good ergonomic performance? Does the interface that not meet the ergonomic requirements also not to be beautiful?

**Figure 4:**
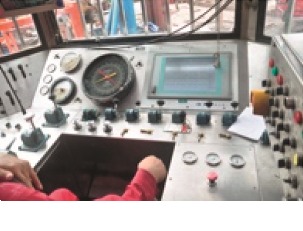
The layout interface simulation of scheme 1.

According to the analysis in Section 6, it can be seen that the human-machine factors are well considered in the layout process of scheme 1. As shown in Figure 4, the driller's visual characteristics are simulated on the platform of CATIA software. A 90 percentile Chinese male human body model is transferred in, with a horizontal distance of 1115 mm between the center of the eyes and the front of the display console, simulating the working state of the male driller. The top left corner of Figure 4 shows the visual field window, which is the picture of the visual field of the human body model. Visual field analysis shows that when the head is in a relaxed state, the whole human-machine interaction interface is within the effective visual area. Weight indicator, as the most frequently observed instrument, is located in the best visual area of human beings, and then other display instruments are arranged to the left and right sides, respectively, based on the center line of the weight indicator, which meets the requirements of human visual characteristics. The scheme 1 not only had the highest aesthetic evaluation value in the four schemes, but also had a good human-machine performance.

In the above schemes, three of the four schemes' (scheme 1: 0.71439, scheme 2: 0.69428, and scheme 4: 0.69496) closeness values to the ideal scheme were very close, and the closeness value of scheme 3 was minimum, that is, 0.07731. The layout objects of scheme 2 and scheme 3 were not arranged according to the frequency of observation, so their ergonomic performance was poor. However, as far as aesthetic design was concerned, the aesthetic design of scheme 2 was good, while that of scheme 3 was the worst. The layout objects of scheme 1 and 4 were laid out according to the ergonomic requirements, and their aesthetic design was also good. It can be seen that a good human-machine interaction interface can realize the coexistence of ergonomic performance and aesthetic requirements. This is consistent with Moshagen's experimental conclusion that visual aesthetics has a positive impact on performance [31]. The layout that does not meet the ergonomic requirements can also have visual aesthetics. Therefore, it is suggested to conduct the human-machine layout design first and then carry out the aesthetic quantitative evaluation.

Third, when extracting aesthetic image factors, Bartlett test of sphericity and KMO test were used to analyze whether the original variables were suitable for factor analysis, and principal component analysis was used to solve the factor loading matrix. These are conventional methods that are widely used. Whether the factor analysis method needs to be improved in the application process remains to be further studied.

Fourth, the layout interface is abstracted as rectangle in the process of aesthetic calculation. This is because the space utilization rate of rectangular is the best, and the rectangular panel conforms to the visual patrol rule of person from left to right, from top to bottom. However, the actual form of human-machine interaction interface is more abundant. The above formula is still applicable to regular geometry such as circle and ellipse. For irregular shape layout interface, it can be abstracted as approximate regular geometry. The research in this paper focuses on theoretical calculation. In the future, we will explore the physiological measurement methods of modern cognitive neuroscience and use the experimental aesthetic means to quantify the aesthetic feeling of interface.

Fifth, the production of aesthetic feeling is that aesthetic subject (user) produces some subjective pleasure in the information exchange with aesthetic object (human-machine interaction interface). Aesthetic cognition has complexity and subjectivity. The aesthetic problem of human-machine interface is affected by many factors. This paper focused on layout, abstracted the form of layout interface and layout objects, and mainly studied the influence of form, location, and size on users' aesthetic experience. The next research will add the influence of color and other factors on aesthetics.

Sixth, the aesthetic degree calculation process of visual aesthetic image factor was relatively complicated. In the future, the computerization of aesthetic degree calculation process will be considered to build an aesthetic degree calculation prototype system. It should provide a visual platform for designers and users to improve the efficiency of layout aesthetic evaluation.

## 8. Conclusion

To quantify the visual aesthetics of human-machine interaction interface layout, according to the research method of Kansei engineering on personal aesthetic preference, the vocabularies used to describe the aesthetic feeling brought to users by interface layout design were collected. Then, the visual aesthetic elements were analyzed by factor analysis method, and six aesthetic image factors that affecting the interface aesthetics were extracted: proportion, conciseness, order, rhythm, density, and equilibrium. The interface aesthetic degree was described by the mathematical language, and six formulas for calculating the aesthetic degree of perceptual images were constructed. Using TOPSIS method, the positive and negative ideal solutions of six aesthetic degree indexes were constructed, and then the distance between the layout interface to be evaluated and the positive and negative ideal solutions was determined, and the comprehensive evaluation value of interface aesthetic degree was obtained. Taking the human-machine interaction interface layout aesthetics evaluation of the driller's console of an AC variable frequency drilling rig as an example, the quantitative evaluation process of visual aesthetics was illustrated. The feasibility and effectiveness of the proposed method were verified by the method of paired comparison commonly used in psychophysics.

## Figures and Tables

**Table 1:** The ten representative samples of human-machine interaction interface.

1	2	3	4	5

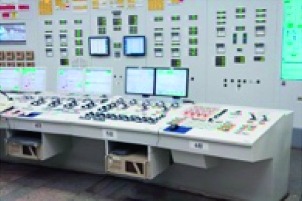	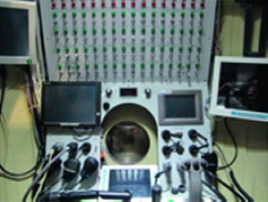	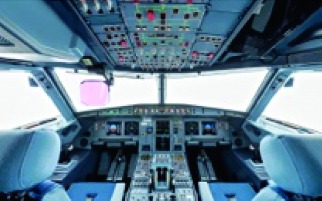	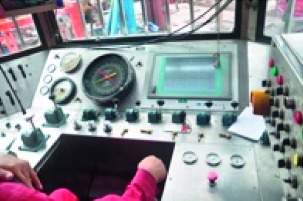	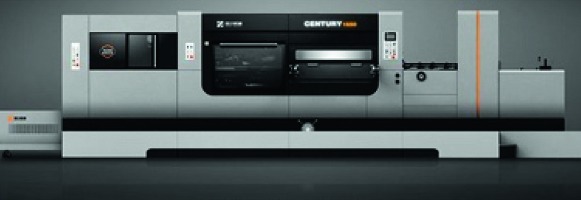
6	7	8	9	10

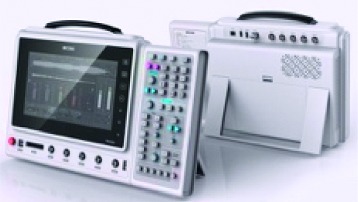	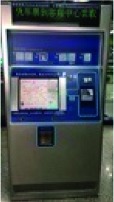	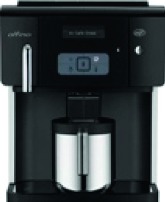	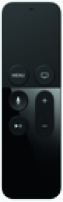	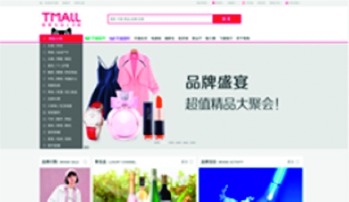

**Table 2:** The factor analysis of visual aesthetic image of interface.

Component	Image factors	Rotation sums of squared loadings		
		Total	% of variance	Cumulative %
1	Proportion	2.685	17.901	17.901
2	Rhythm	2.651	17.675	35.576
3	Equilibrium	2.614	17.428	53.004
4	Density	2.380	15.865	68.869
5	Conciseness	1.567	10.448	79.318
6	Order	1.149	7.663	86.980

**Table 3:** Visual aesthetic image structure of interface.

Image factors	Image vocabularies
Proportion	Golden section, uniformity, scale, proportional aesthetic feeling
Conciseness	Simple, concise, tidy, modular, unified, easy to use, clear
Order	Sequence, ease, reliability, priority, guidance
Rhythm	Harmony, cycle, echo, rhythmical, regularity, metrical sense, cadence
Density	Concentration, dense, compactness, centrality, relaxation
Equilibrium	Balance, symmetry, stability, coordination, sense of volume

**Table 4:** Layout objects and parameters.

Layout objects	Width (mm)	Height (mm)
Parameters instrument	590	500
Weight indicator	360	360
Stand pipe pressure, rotary torque, crabs torque	192	192
Hydraulic pressure	130	130
Left clamp pressure, right clamp pressure, safety clamp pressure, air pressure, rotary oil pressure, winch oil pressure	100	100

The layout interface of scheme 1, scheme 2, and scheme 3: 1700 × 560		
The layout interface of scheme 4: 1850 × 560		

**Table 5:** The index values of aesthetic degree of each scheme.

Image factors	*M*_1_ proportion	*M*_2_ conciseness	*M*_3_ order	*M*_4_ rhythm	*M*_5_ density	*M*_6_ equilibrium
Scheme 1	0.81670	0.34722	0.75000	0.45897	0.85732	0.69968
Scheme 2	0.81670	0.34722	0.75000	0.45297	0.85732	0.69968
Scheme 3	0.81670	0.34167	0.75000	0.36705	0.85732	0.53646
Scheme 4	0.78991	0.34722	0.75000	0.49936	0.94997	0.60830

**Table 6:** The weight of aesthetic degree evaluation index.

Image factors	*M*_1_ proportion	*M*_2_ conciseness	*M*_3_ order	*M*_4_ rhythm	*M*_5_ density	*M*_6_ equilibrium
Weight	0.17901	0.10448	0.07663	0.17675	0.15865	0.17428

**Table 7:** The sequence of picture presentation.

	A	B	C	D
A	^*∗*^	12	8	7
B	1	^*∗*^	11	9
C	5	2	^*∗*^	10
D	6	4	3	^*∗*^

**Table 8:** Comparison and selection of layout schemes by subjects.

Trial	Stimuli	Selection				
		Subject 1	Subject 2	Subject 3	Subject 4	Subject 5
1	AB	A	A	A	A	A
2	BC	B	C	B	C	B
3	CD	D	D	D	D	D
4	BD	D	D	D	D	D
5	AC	A	A	A	A	A
6	AD	D	A	A	D	A
7	DA	A	A	A	D	D
8	CA	A	A	A	A	A
9	DB	D	D	B	D	D
10	DC	D	D	D	D	D
11	CB	B	B	C	C	B
12	BA	A	A	A	A	A

**Table 9:** Paired comparison data collation of layout schemes.

	A	B	C	D
Subject 1	5	2	0	5
Subject 2	6	1	1	4
Subject 3	6	2	1	3
Subject 4	4	0	2	6
Subject 5	5	2	0	5
Total	26	7	4	23
Order	1	3	4	2

## Data Availability

The data used to support the findings of this study are available from Li Deng (dengli@swpu.edu.cn) upon request.
